# Classification Scheme for Arm Motor Imagery

**DOI:** 10.1007/s40846-016-0102-7

**Published:** 2016-01-29

**Authors:** Mojgan Tavakolan, Xinyi Yong, Xin Zhang, Carlo Menon

**Affiliations:** School of Engineering Science, Simon Fraser University, Burnaby, BC V5A 1S6 Canada

**Keywords:** Pattern recognition, Feature extraction, Brain computer interface (BCI), Support vector machine (SVM)

## Abstract

Facilitating independent living of individuals with upper extremity impairment is a compelling goal for our society. The degree of disability of these individuals could potentially be reduced by using robotic devices that assist their movements in activities of daily living. One approach to control such robotic systems is the use of a brain–computer interface, which detects the user’s intention. This study proposes a method for estimating the user’s intention using electroencephalographic (EEG) signals. The proposed method is capable of discriminating rest from various imagined arm movements, including grasping and elbow flexion. The features extracted from EEG signals are autoregressive model coefficients, root-mean-square amplitude, and waveform length. Support vector machine was used as a classifier, distinguishing class labels corresponding to rest and imagined arm movements. The performance of the proposed method was evaluated using cross-validation. Average accuracies of 91.8 ± 5.8 and 90 ± 4.1 % were obtained for distinguishing rest versus grasping and rest versus elbow flexion. The results show that the proposed scheme provides 18.9, 17.1, and 16.5 % higher classification accuracies for distinguishing rest versus grasping and 21.9, 17.6, and 18.1 % higher classification accuracies for distinguishing rest versus elbow flexion compared with those obtained using filter bank common spatial pattern, band power, and common spatial pattern methods, respectively, which are widely used in the literature.

## Introduction

In recent years, the use of brain–computer interfaces (BCIs) has been shown to be promising for detecting the users’ intention and controlling robotic devices [1]. A BCI system detects electrical changes in the brain and attempts to find patterns in these changes that are related to specific movements or thoughts. Several non-invasive and invasive methods have been proposed to detect these patterns [2]. In this study, non-invasive electroencephalography (EEG)-based BCIs are of particular interest.

EEG signals can be correlated to tasks performed by an individual [3]. Such tasks include mental computation [4], imagining motor movements [5], imagining speech [6], and experiencing emotions [7]. Various classification methods have been proposed for classifying EEG signals. For example, the Elman neural network (ENN) trained by the resilient backpropagation (BP) algorithm was used for the classification of mental tasks, with an accuracy of 86 % obtained [8]. The extracted power of the spectral frequencies has been used for the classification of five mental tasks using a fuzzy classifier [9], with a classification efficiency of 65–100 % obtained. Empirical mode decomposition has been used for feature extraction [10]. An accuracy of 91 ± 5 % was obtained when linear discriminant analysis (LDA) was used and an accuracy of 87 ± 5 % was achieved when a multilayer perceptron (MLP) network was implemented. MLP–BP with adaptive autoregression [11] achieved an accuracy of 81.80 %.

**Fig. 1:**
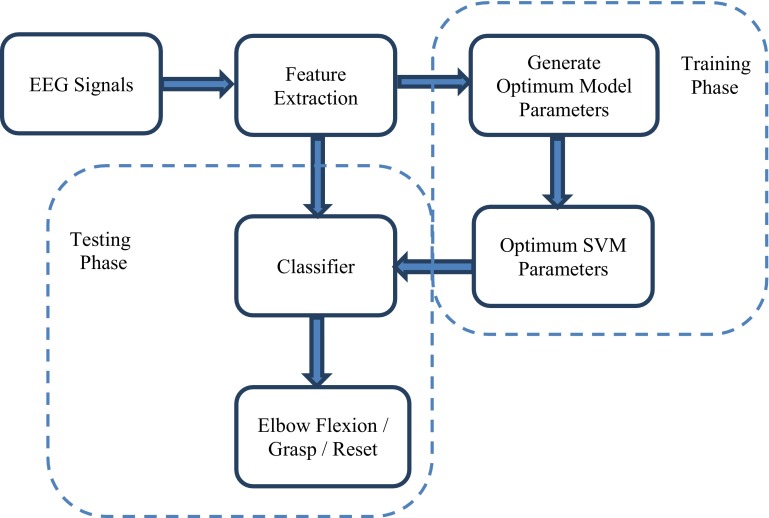
Proposed classification scheme

The detection of the task the user intends to perform is still a challenge. The present study proposes a pattern recognition scheme (Fig. 1) to extract the patterns of specific upper extremity (UE) imagined motor movements from acquired EEG signals. Measuring brain activity through EEG signals creates a large amount of data. Feature extraction highlights important data and eliminates redundant or non-informative data by transforming collected signals into a feature vector. This transformation causes a dimensionality reduction, which facilitates the classification process. Time-domain features are computed based on the signals’ amplitudes, and require no transformation or complex calculation [12]. Time-domain features have low computational complexity and are considered as an appropriate option for real-time BCI systems [13]. Therefore, time-domain features such as autoregressive (AR) model coefficients, root-mean-square (RMS) amplitude, and waveform length (WL) are employed in this study. The EEG patterns corresponding to the imagined motor movements are extracted using pattern recognition techniques. The performance of the proposed algorithm was compared to that of three widely used EEG pattern recognition methods. The proposed method outperformed these methods. The proposed EEG classification scheme was designed to be potentially suitable for controlling robotic devices that assist individuals with an impaired UE. This study is a reference for further enhancement of the recognition rate of EEG patterns and making BCIs more practical.

## Protocols and Data Collection

Protocols were defined to simulate simple activities of daily living involving the arm. The identified protocols considered a combination of several imagined arm movements, including grasping, flexing the elbow, and rest. Motor imagery of grasping and elbow movements is suitable for controlling robotic exoskeletons and for assistance and rehabilitation of UEs [14, 15]. For example, the user could imagine moving their elbow to control a robotic device and receive assistance to extend their arm towards a cup, and then imagine grasping to receive assistance in grasping the cup for drinking [14, 15].

**Fig. 2:**
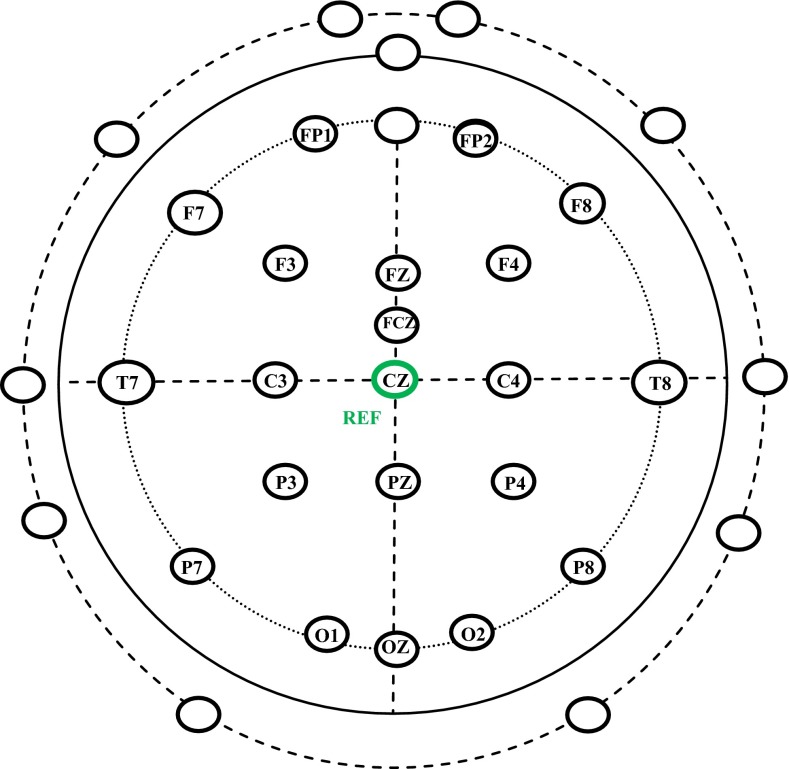
EEG electrode positions employed in this study

The protocol involved non-invasive EEG data recording using the Geodesic sensor net (Electrical Geodesics, Inc., Eugene, OR, USA). The 32-channel Geodesic sensor net was applied to the participant’s head [16]. The locations of electrodes are presented in Fig. 2. The labeled electrodes were employed for the BCI system. The unlabeled electrodes were not considered in this study because they were very close to sources that generate muscle activity or artifacts. The vertex Cz position in Fig. 2 was used as a reference.

Twelve healthy volunteers participated in this study. Each volunteer signed a consent form. This study was approved by the Office of Research Ethics, Simon Fraser University. The approval number is 2012s0527. The volunteers started the experiment with the imagined arm in the rest position. Protocols P1, P2, and P3 were used to extract data for classification. In protocol P1, the volunteer was asked to imagine the arm in the rest position. In protocol P2, the volunteer was asked to imagine applying a comfortable force while grasping. In protocol P3, the volunteer was asked to imagine lifting the arm. Each volunteer received a visual command on the monitor for the task they were asked to perform. Each experiment lasted 1.5 h. The experiment consisted of four sessions, each of which lasted 12 min. The participant was asked to perform each designated task for 3 s, followed by 5–7 s of rest. The data was amplified and sampled at 1000 Hz using a Geodesic Net Amps 400 series amplifier (Electrical Geodesics, Inc.) [17]. The EEG data were transmitted via the TCP/IP protocol to the computer. Throughout the experiment, the electrode impedance was maintained at below 50 kΩ. The participants could take a break whenever needed.

## Materials and Methods

### CSP, FBCSP, and Band Power Approaches

A number of approaches have been proposed for estimating motor imagery EEG [18, 19]. Among these methods, the common spatial pattern (CSP) [20] method seems to be the most effective, yielding the best BCI performances in calculating spatial filters for detecting EEG patterns [21]. The CSP method maximizes the variance of the spatially filtered signals for one class while minimizing it for the other class for distinguishing features. The CSP method is suitable for EEG-based BCIs [22].

The filter bank CSP (FBCSP) method is also very effective, yielding high BCI performances [23]. The first stage employs a filter bank to filter EEG signals into multiple frequency bands. The second stage performs spatial filtering using the classical CSP method. Among the multiple spatial filters obtained, the best resulting features are selected using feature selection algorithms [23].

Band power is another successful method. The logarithmic band power is based on the design of the original Graz BCI [24]. The band power method is often used in BCI pattern recognition [25].

The classification scheme was performed using the CSP, FBCSP and band power methods [22, 26]. The collected data are band-pass-filtered from 6 to 40 Hz using an FIR filter to reduce interference from other sources. Then, the features are extracted by applying CSP, FBCSP, and band power methods. The obtained features are used to train a linear discriminator [27]. 10-by-tenfold cross-validation was used for performance validation.

### Proposed Method

Time-domain features such as AR model coefficients, RMS amplitude, WL are extracted by the proposed method. RMS amplitude and WL provide one feature for each channel of EEG signals. AR models provide four features for each channel of EEG signals. The proposed pattern recognition scheme is performed off-line. The features are calculated by segmenting the collected EEG signal into 250-ms intervals and then calculating a set of features for each segment.

AR model coefficients provide information regarding previous samples. The current value is predicted based on the previous output values. The AR models are linear combinations of previous samples. The current value *t*_*n*_ is expressed as:

1 $$t_{n} = \sum\limits_{i = 1}^{p} {a_{i}^{p} t_{n - i} }$$

where {*a*_*i*_ for *i* = 1,…, *p*} are AR model coefficients and *p* is the order of the AR model.

The RMS amplitude feature provides information regarding the amplitude of the EEG signal. This feature is computed as:

2 $$RMS_{r} = \sqrt {\frac{{r_{1}^{2} + r_{2}^{2} + \cdots + r_{n}^{2} }}{n}}$$

where *r*_*i*_ is the amplitude of the *i*th sample and *n* is the number of samples.

The WL feature is a measure of the waveform complexity in each segment. WL is represented as:

3 $$y = \sum\limits_{i = 1}^{N - 1} {\left| {w_{i + 1} - w_{i} } \right|}$$

where *w*_*i*_ is the amplitude of the *i*th sample and *N* is the number of samples.

To achieve good classification performance, the set of input features and the choice of the applied classifier are crucial [28]. The support vector machine (SVM), an efficient and accurate classifier with relatively low complexity, is used here. The main idea behind SVM [29] is to find discriminant hyperplanes that separate the data that belong to different classes with the maximum possible margin. Maximizing the margins increases the generalization capabilities of the classifier. In its general formulation, SVM requires solving the following optimization problem:

4 $${\text{Min}}\,\frac{1}{2}\left\| a \right\|^{2} \,+\, c\sum\limits_{i = 1}^{N} {\xi_{i} }$$

5 $${\text{Subject to }}w_{i} y\left( {x_{i} } \right) \ge 1 - \xi_{i} \quad{\text{where }}i = 1 \cdots N\quad {\text{and}}\,\xi_{i} \ge 0$$

where *y* is the learned model, *c* > *0* is the penalty factor, *a* is the vector representing adaptive model parameters, *w*_*i*_ is the label associated with a data point, *i* is the index associated with a data point, *ξ*_*i*_ is the slack variable, *x*_*i*_ is the vector representing a data point, and *N* is the number of data points.

SVM works well in high-dimensional spaces. SVM maps the data to higher-dimensionality space with the help of a kernel function [29]. The radial basis function (RBF) was selected as the kernel function. The RBF kernel has the fewest hyper-parameters, which reduces the complexity of the pattern recognition model. The mathematical representation of the RBF kernel is:

6 $$k\left( {y_{i} ,y_{j} } \right) = \exp \left( { - \gamma \left\| {y_{i} - y_{j} } \right\|^{2} } \right)$$

where *γ* is the kernel parameter and $y_{i} , y_{j}$ are training vectors.

The goal was to find the optimal kernel parameters so that the classifier could accurately predict the user’s intention. Tenfold cross-validation was used here to prevent the over-fitting problem. In tenfold cross-validation, the dataset was first divided into 10 subsets of equal size. Each subset was then sequentially tested (testing phase) using the classifier trained on the remaining 9 subsets (training phase). A grid search along with tenfold cross-validation was used for the classifier parameters. Various values were tested and those that did not over-fit the data and gave the highest cross-validation accuracy were selected as the optimal kernel parameters. Those that gave the lowest cross-validation accuracy were selected as the non-optimal parameters.

## Results

**Fig. 3:**
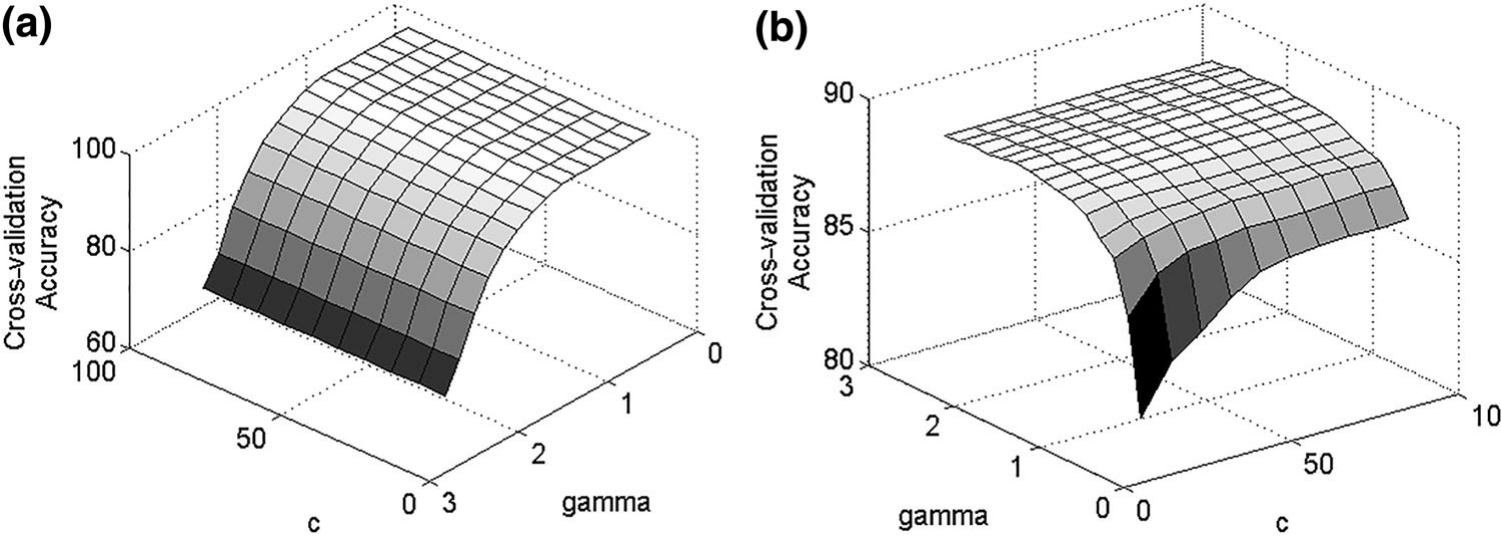
Cross-validation accuracies based on *c* and *γ* parameters. **a** Imagined grasping and rest. **b** Imagined elbow flexion and rest

The optimal values for the kernel parameters were selected according to the highest value of the cross-validation accuracy for each individual. The obtained optimal kernel parameters were then used to build a model for classifying the imagined arm movements. Figure 3 shows the obtained results for the optimal kernel parameters for classifying imagined grasping and elbow flexion versus rest for a single participant. As shown, the highest cross-validation accuracy occurred in the interval (0, 3) for *γ* and (0, 100) for *c*. These intervals were selected for the identification of the optimal kernel parameters for all participants.

**Fig. 4:**
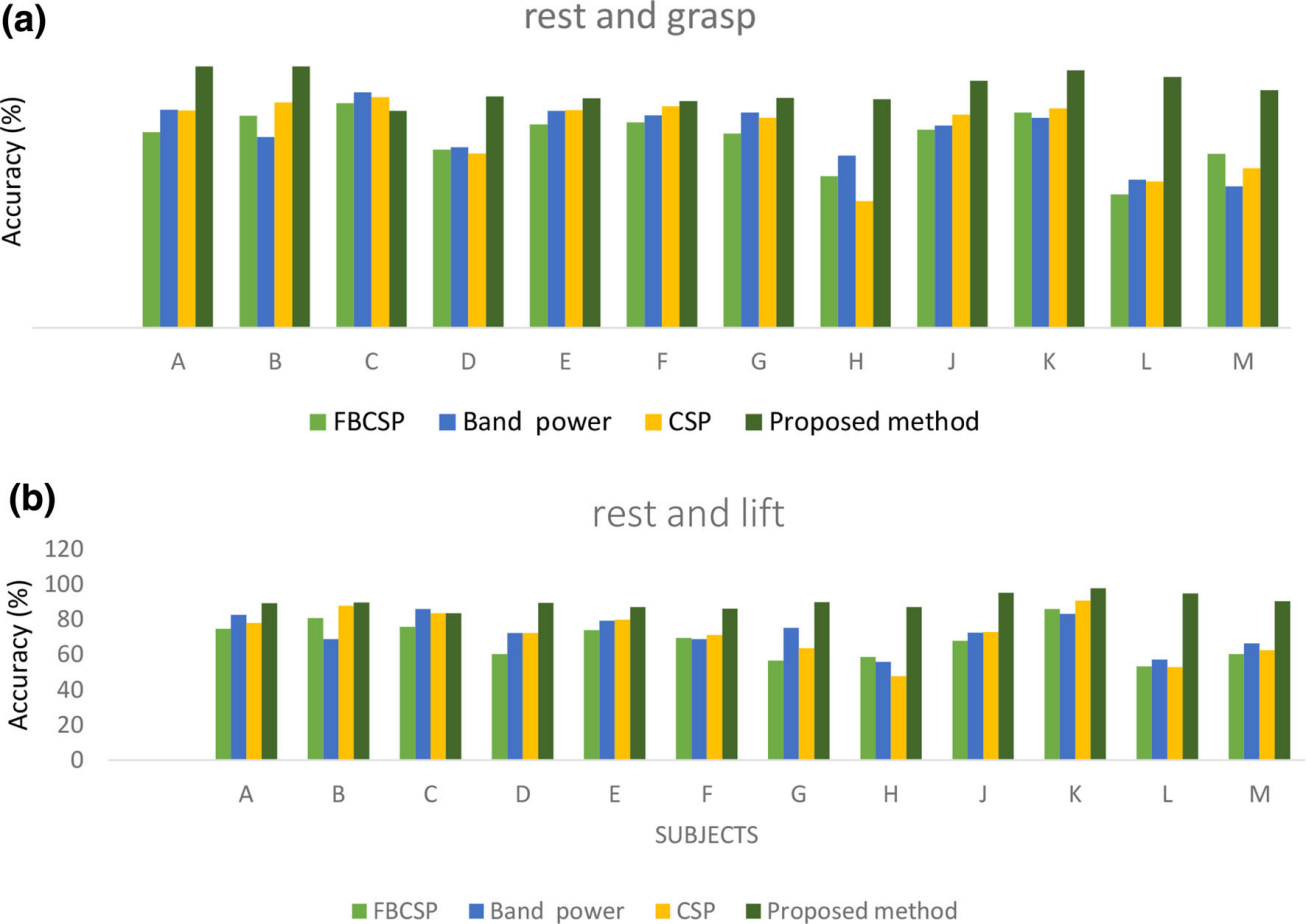
Classification accuracies of proposed, CSP, FBCSP, and band power methods for each individual. **a** Imagined grasp and rest. **b** Imagined elbow flexion and rest

**Fig. 5:**
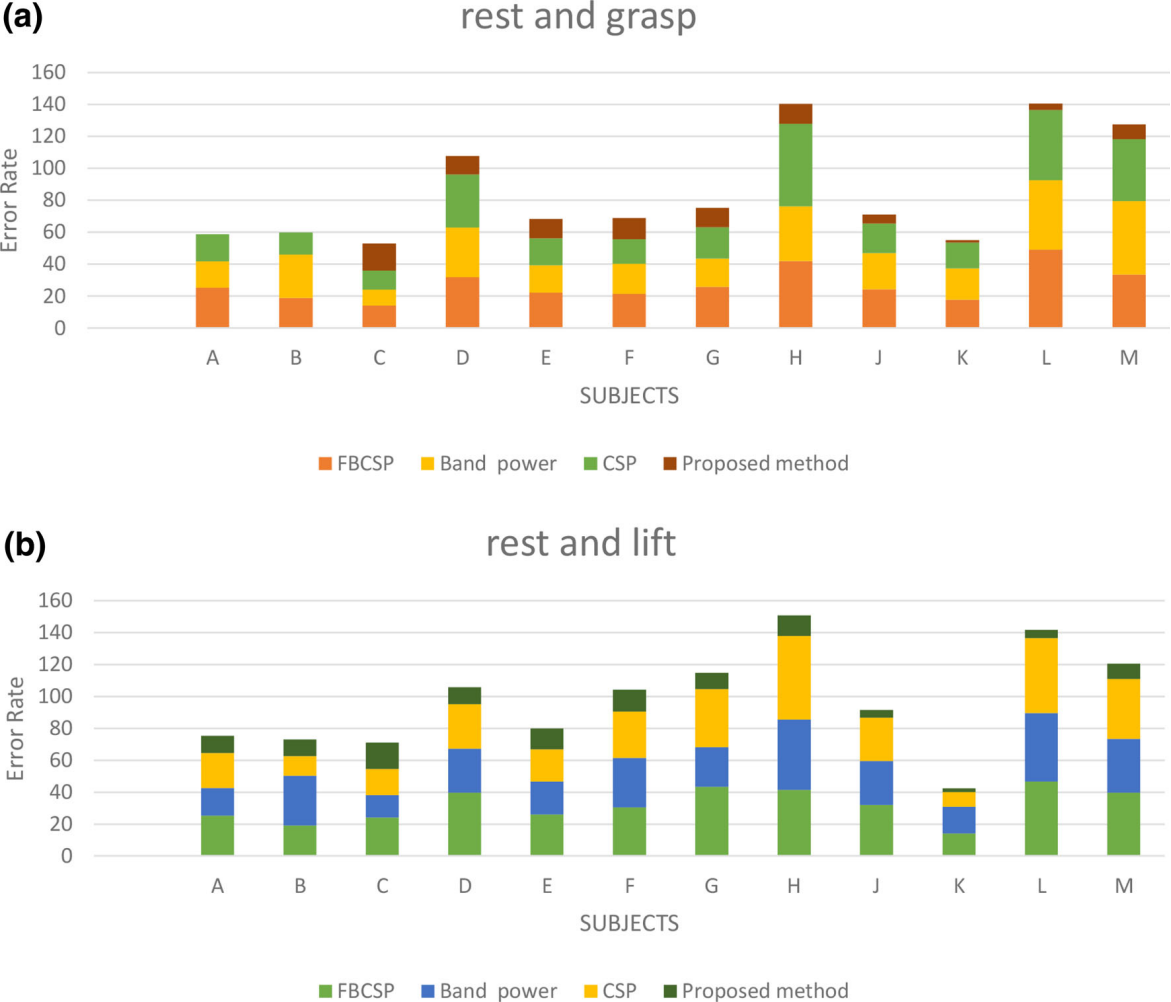
Cumulative error rates of proposed, CSP, FBCSP, and band power methods for each individual

The obtained optimal kernel parameters and the proposed method classification accuracies are presented in Tables 1 and 2, respectively, for each of the twelve volunteers (denoted as A–M, respectively). These selected parameters were then used to build the optimal pattern recognition model for each individual. The reported proposed classification accuracy for each volunteer is the percentage of data which were correctly classified. The pattern recognition accuracies and cumulative error rates obtained using the optimal model of the proposed method, CSP, FBCSP, and band power methods are presented in Figs. 4 and 5, respectively, for each individual.

**Table 1:** Proposed method classification accuracy and optimal model parameters, *c* and *γ,* for classifying rest versus grasping

Subject	Rest-grasping optimal parameters *c* and *γ*	Accuracy (%)
A	10, 0.2	100
B	10, 0.2	100
C	10, 2.3	83
D	10, 1.9	88.5
E	15, 1.7	87.8
F	15, 1.1	86.7
G	10, 1.4	87.9
H	10, 1.9	87.4
J	10, 1.6	94.4
K	70, 1	98.5
L	90, 1	95.9
M	10, 2.5	90.8

**Table 2:** Proposed method classification accuracy and optimal model parameters, *c* and *γ,* for classifying rest versus elbow flexion

Subject	Rest-elbow optimal parameters *c* and *γ*	Accuracy (%)
A	10, 1.7	89.3
B	90, 0.4	89.6
C	10, 1.6	83.6
D	10, 1.5	89.4
E	10, 2.2	87
F	15, 1.1	86.2
G	10, 2	89.9
H	10, 2.3	87
J	10, 2	95.2
K	35, 2.3	97.7
L	30, 2.1	94.9
M	10, 1.4	90.3

**Fig. 6:**
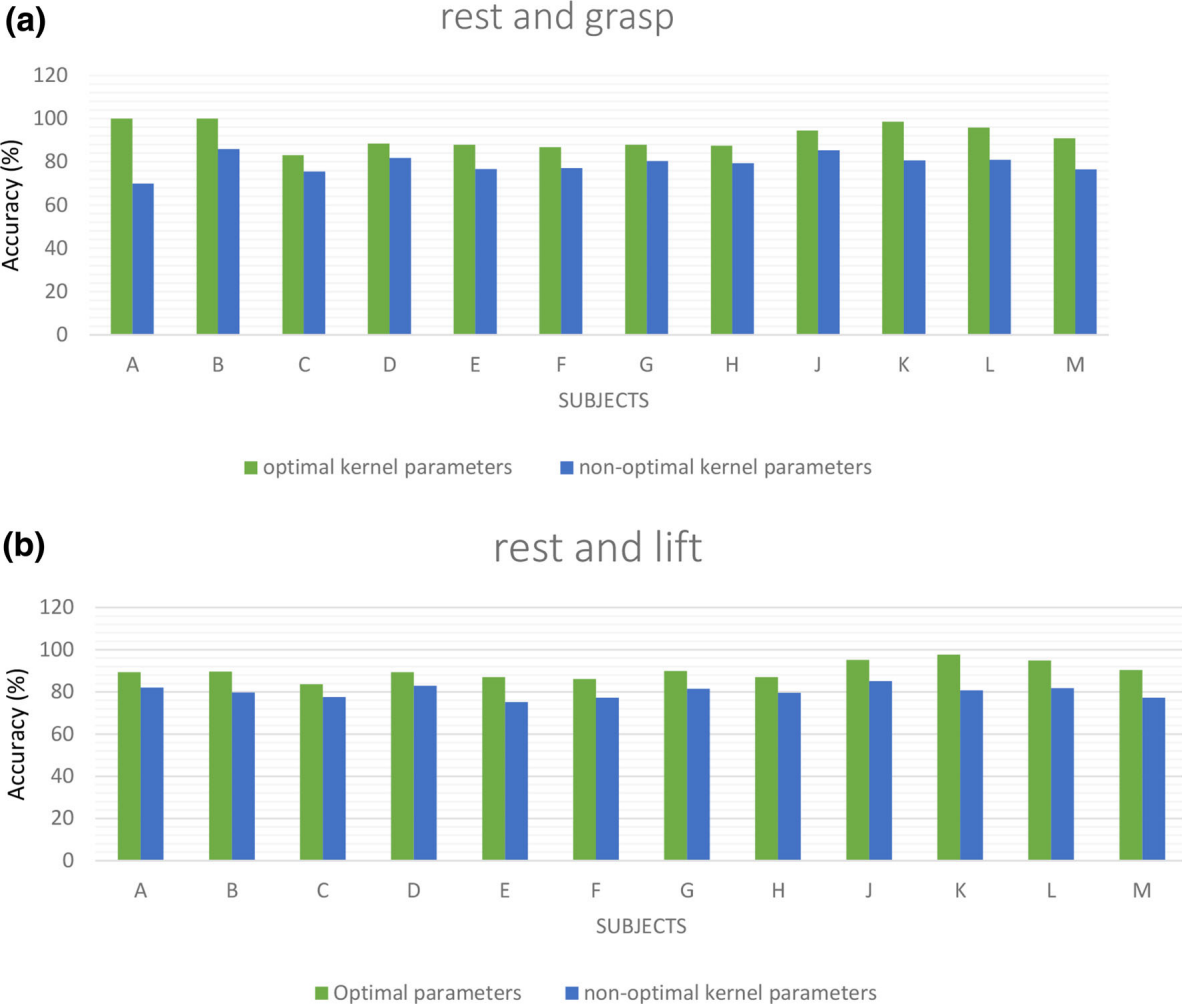
Classification accuracies of proposed method using optimal and non-optimal parameters for each individual. **a** Imagined grasping and rest. **b** Imagined elbow flexion and rest

**Fig. 7:**
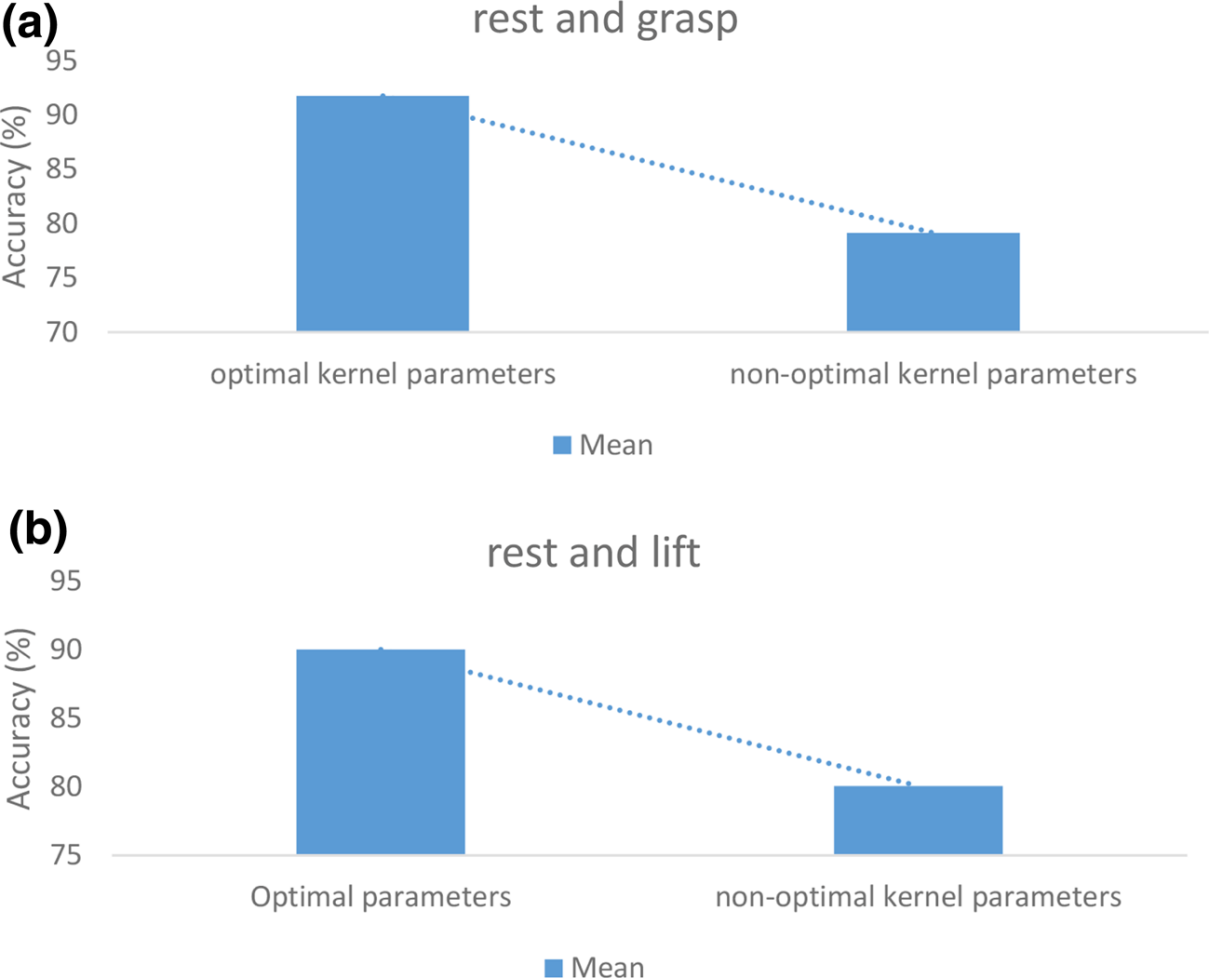
Average classification accuracies of proposed method using RBF kernel for optimal and non-optimal parameters. **a** Imagined grasping and rest. **b** Imagined elbow flexion and rest

Figure 6 compares the optimal and non-optimal parameters for each individual. As shown, the pattern recognition accuracy of the RBF kernel function with optimal parameters was higher compared to that of the RBF kernel function with non-optimal parameters for all subjects. The pattern recognition rate increased by more than 9 % on average for identifying imagined grasping and elbow flexion patterns versus the rest pattern when the optimal parameters were used (see Fig. 7).

**Fig. 8:**
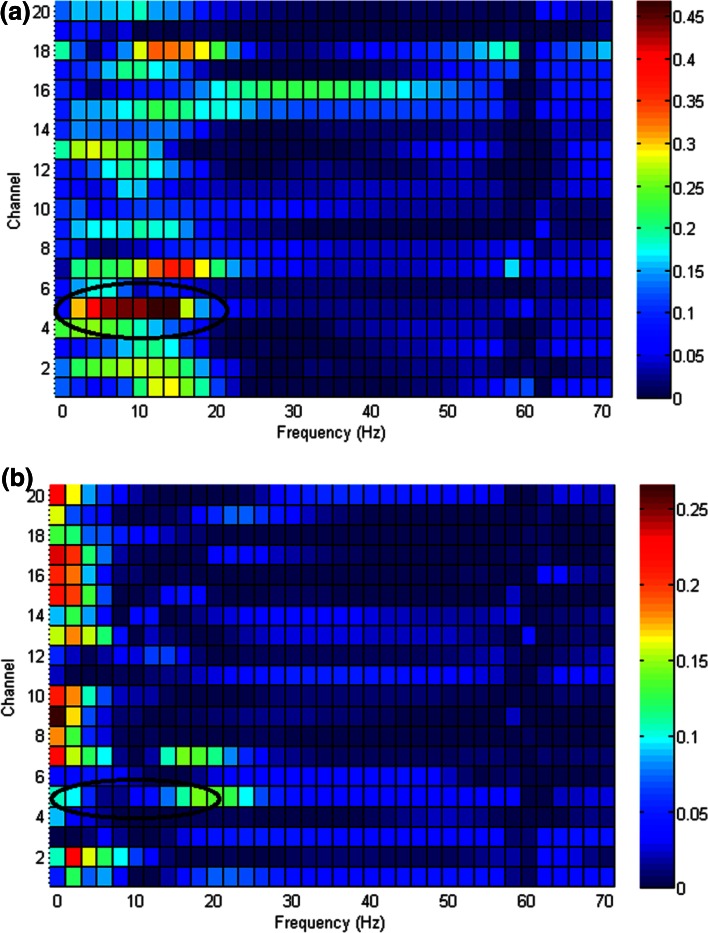
Variation plot for imagined arm movements and rest. Electrodes corresponding to Channels 1–20 are Fp1, Fp2, F3, F4, C3, C4, P3, P4, O1, O2, F7, F8, T7, T8, P7, P8, Fz, Nas, Pz, and Oz, respectively. **a** Imagined grasping and rest for Subject A. **b** Imagined elbow flexion and rest for Subject F

The overall results obtained for the proposed method indicate that it is acceptable and promising. 100 % accuracy was obtained for subjects A and B for the imagined grasping and rest. Accuracies of over 90 % were obtained for subjects J, K, L, and M. A large variation in the brain signal between imagined grasping and rest occurred for subject A over the sensorimotor cortex, resulting in the high classification accuracy for subject A. A small variation in the brain signal between imagined elbow flexion and rest occurred for subject F over the sensorimotor cortex, resulting in a low pattern recognition accuracy (Fig. 8).

**Fig. 9:**
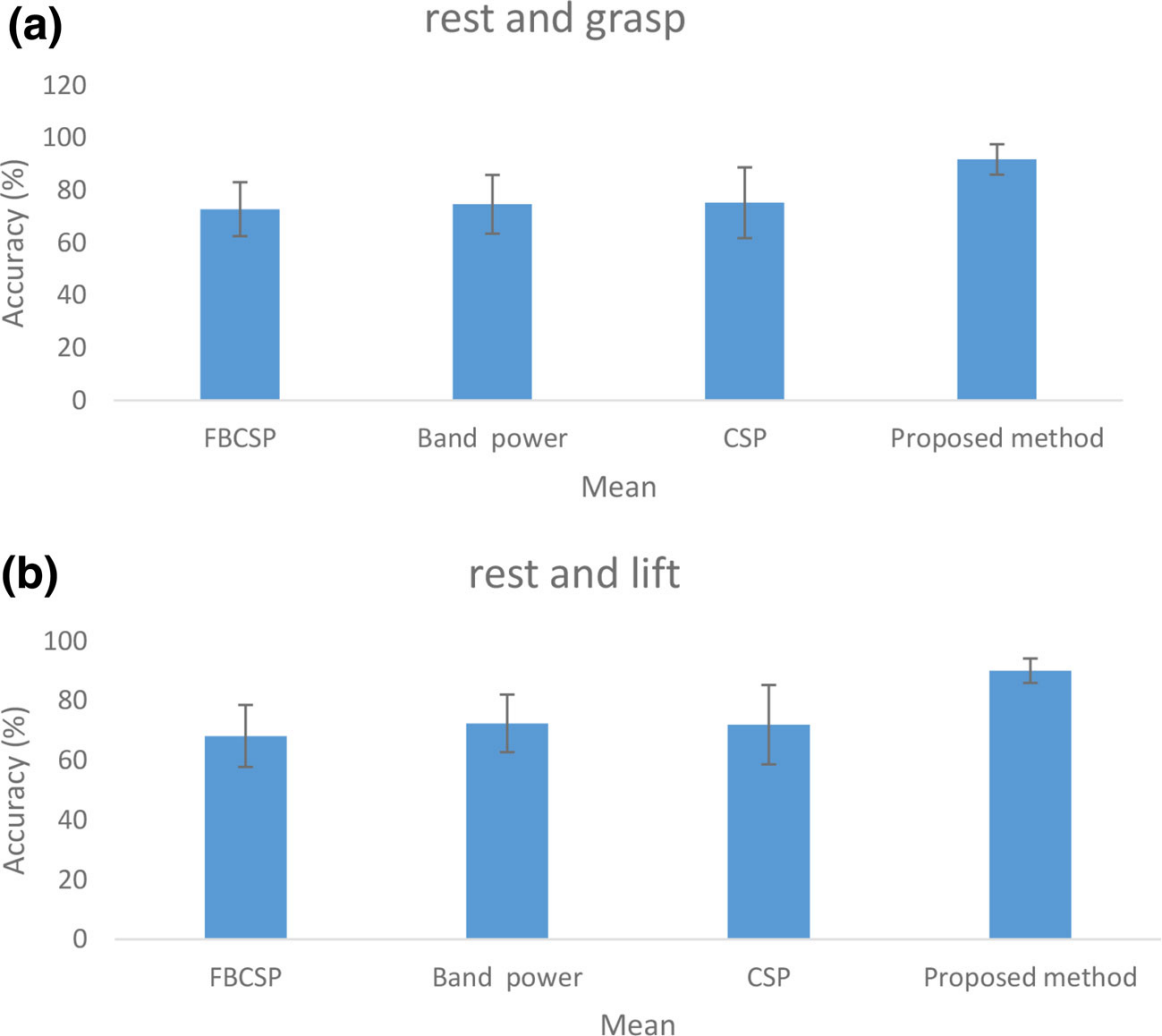
Average classification accuracies of proposed, CSP, FBCSP, and band power methods. **a** Imagined grasping and rest. **b** Imagined elbow flexion and rest

Figure 9 shows the average classification accuracies for each method. The patterns corresponding to grasping, rest, and elbow flexion for imagined arm movements were accurately identified. The average classification accuracy for the proposed method is higher compared to those of the CSP, FBCSP, and band power methods. The analysis of variance results show that there were statistically significant differences (*p* < 0.015) between the results obtained using the proposed method and those obtained using the other methods. The average classification accuracy results indicate that the CSP, FBCSP, and band power methods are all powerful and that there is a small difference their pattern recognition performance.

## Discussion

Optimizing the kernel parameters was the key factor in improving the performance of the proposed method. It was demonstrated that on average the performance of the RBF kernel function with optimal parameters was higher compared to that with non-optimal parameters.

There were relatively low classification error rates for subjects A and B using the CSP, FBCSP, and band power methods for imagined grasping and rest classification. However, the error rate was zero for these volunteers using the proposed optimal model. For subjects H, L, and M, there were high classification error rates using the CSP, FBCSP, and band power methods. In contrast, the overall error rates were acceptable for these volunteers using the proposed optimal model, which shows that for BCI applications, these features and classifier are a potential option.

In this study, which was designed to assess the performance of the proposed classification scheme, the patterns of imagined grasping, elbow flexion, and rest were successfully recognized. An acceptable classification performance (error rate of below 10 %) was obtained for the classification of arm motor imagery using the proposed method. The data were reasonably separable and well modeled by the extracted features and optimal SVM model. According to the obtained results, the RBF kernel function and the set of extracted features are suitable for the pattern recognition of imagined arm movements.

## Conclusion

The possibility of associating EEG patterns with the imagining of arm movements was investigated. Our results support the hypothesis that successful pattern recognition can be achieved when discriminating imagined arm movements of users in vital activities of daily living. The identified classes in this research were imagined grasping, rest, and elbow flexion. The SVM classifier was shown to be suitable for discriminating the rest state from two imagined arm movements of volunteers.

Selecting optimized kernel function parameters and appropriate features was the key factor to obtaining satisfactory recognition results. The AR model coefficients, RMS amplitude, and WL were extracted to identify patterns in the acquired EEG signals. The implemented pattern recognition strategy was able to identify various imagined arm movements with superior performance compared to those of the CSP, FBCSP, and band power methods.

In future work, it would be interesting to investigate the feasibility of using the pattern recognition of EEG signals to estimate UE imaginary motor tasks in individuals with neurological disorders, including individuals with stroke. In addition, it would be interesting to conduct online experiments to validate that acceptable performance can be obtained.
